# Incidence, predictors and health outcomes of delirium in very old hospitalized patients: a prospective cohort study

**DOI:** 10.1186/s12877-022-02932-9

**Published:** 2022-03-29

**Authors:** Meng Zhang, Xuemei Zhang, Langli Gao, Jirong Yue, Xiaolian Jiang

**Affiliations:** 1 West China School of Nursing, Sichuan University/ Department of Geriatrics and National Clinical Research Center for Geriatrics, West China Hospital, Sichuan University, Chengdu, Sichuan Province China; 2 Department of Geriatrics and National Clinical Research Center for Geriatrics, West China Hospital, Sichuan University, Chengdu, Sichuan Province China; 3 West China School of Nursing, Sichuan University, Chengdu, NO. 37 GuoXue Road, Chengdu, 610041 Sichuan province China

**Keywords:** Delirium, Risk factor, Cognitive impairment, Prediction model, Very old patients

## Abstract

**Background:**

Delirium is a common complication that leads to poor health outcomes in older patients undergoing treatment. Due to severe consequences, early recognition of high-risk patients and risk factors for delirium are crucial in the prompt initiation of prevention measures. However, research in medically hospitalized patients aged ≥80 years remains limited. This study aimed to determine the incidence, predictors and health outcomes of delirium in very old (aged ≥80 years) hospitalized patients in China.

**Methods:**

A prospective study was conducted in individuals aged ≥80 years admitted to geriatric departments. Potential risk factors were assessed within 24 h after hospital admission. Screening for delirium was performed on admission and every 48 h thereafter for 14 days and assessed if acute mental status changes were observed. During hospitalization, health outcomes were recorded daily.

**Results:**

Incident delirium occurred in 109 of 637 very old hospitalized patients (17.1%). The independent predictors of delirium in hospitalized patients aged 80 and over were cognitive function impairment [OR 17.42, 95% CI:(7.47–40.64)], depression [OR 9.30, 95% CI: (4.59–18.84)], CCI ≥ 5 [OR 4.21, 95% CI: (1.48–12.01)], sleep deprivation [OR 3.89, 95% CI: (1.71–8.82)], infection [OR 3.33, 95% CI: (1.70–6.54)], polypharmacy (≥5 medications) [OR 2.85, 95% CI: (1.51–5.39)], constipation [OR 2.58, 95% CI: (1.33–5.02)], and emergency admission [OR 2.13, 95% CI: (1.02–4.45)]. Patients with delirium had significantly longer hospital stays(*P* < 0.001) and higher percentages of physical restraint use(*P* < 0.001) and falls (*P* = 0.001) than those without delirium,.

**Conclusion:**

The incidence of delirium was high in hospitalized patients aged ≥80 years admitted to the geriatric department and was associated with prolonged hospital stay and higher rates of physical restraint use and falls. In this population, the most important independent risk factors for incident delirium were cognitive function impairment and depression. Health care professionals should recognize and initiate interventions for delirium early in geriatric patients.

**Supplementary Information:**

The online version contains supplementary material available at 10.1186/s12877-022-02932-9.

## Background

Delirium, a geriatric syndrome, is characterized by acute changes in attention, awareness and cognition and is caused by a medical condition that cannot be better explained by a pre-existing neurocognitive disorder [1]. It is extremely common in geriatric, postsurgical, intensive care unit (ICU), and palliative care patients [2–5]. Among older medical patients aged ≥65 years, the incidence of delirium ranges from 20 to 29% [2]. The absolute number may increase with the aging of the population. Delirium can contribute to prolonged hospitalization; functional deterioration; and increased rates of protracted mortality, dementia and hospital-associated adverse events (falls, pressure ulcers, and unplanned extubation) [2, 6, 7]. In addition, estimates of total healthcare costs related to delirium are more than $164 billion per year in the United States [8].

Despite the high incidence and poor prognosis of delirium, it has been demonstrated that approximately 30% ~ 53% of cases can be prevented [9, 10]. Early identification of risk factors and measures targeting these factors are critical first steps in the development of effective preventive strategies [11]. Delirium is frequently multifactorial in the elderly, dependent on complex interactions between vulnerable patients with several predisposing factors and exposure to precipitating factors [11, 12].

Currently, several studies have systematically examined the risk factors for delirium in older hospitalized patients [13–19]. However, these studies mainly analysed Caucasian populations. Ethnographic discrepancies may result in different outcomes. Although there have been studies on delirium in older Chinese people, the majority of them focused on those in postoperative, ICU, terminal malignancy, or subacute medical settings [20–23] and therefore are not broadly applicable to the general medical population, which is particularly susceptible to developing delirium. Recently, a study in medically hospitalized Chinese patients determined the risk factors for prevalent delirium [24]. Nevertheless, incident delirium can also be a major challenge, especially for delirium prevention. A delirium predictor study that targets older Chinese adults aged 80 years and over, which has been demonstrated to be an important cut-off for incident delirium, has never been performed [25]. Prompt recognition of delirium is important for the initiation of effective preventive treatment as soon as possible. Therefore, the identification of potential risk factors among older medical inpatients is crucial.

The present study aimed to determine the incidence and identify the predictors of delirium in very older (aged ≥80 years) persons admitted to a medical ward in China. Furthermore, we aimed to contrast the clinical outcomes of patients with and without delirium.

## Methods

### Design, setting and participants

This prospective cohort study was conducted in four geriatric departments of West China Hospital of Sichuan University from June 2016 to May 2017. Patients aged ≥80 years who were admitted to the geriatric department and who had an anticipated length of stay longer than 2 days were eligible for inclusion. The exclusion criteria included (1) delirium on admission assessed using the Confusion Assessment Method (CAM); (2) inability to communicate due to severe dementia, legal blindness, or severe deafness; (3) a terminal condition with a life expectancy < 6 months; and (4) a documented history of schizophrenia or psychosis. This study was approved by the Research Ethics Committee of West China Hospital of Sichuan University (#201440).

### Determination of delirium and subtypes

Incident delirium was defined as delirium that was not present on enrolment but developed during hospitalization. The 3-min Diagnose Interview for CAM-defined Delirium (3D-CAM) was used to determine delirium in our study [26]. This tool includes 20 items that streamline the evaluation of the 4 CAM diagnostic features: (1) acute change and fluctuating course; (2) inattention; (3) disorganized thinking; and (4) altered level of consciousness. Delirium was considered to exist when features (1) and (2) were both present, and at the same time, either features (3) or (4) (or both) were present. The Chinese 3D-CAM has 94.73% sensitivity and 97.92% specificity for the diagnosis of delirium in older Chinese patients [27]. If delirium was diagnosed, the Delirium Rating Scale-Revised-98 was used to classify hyperactive, hypoactive, or mixed subtypes of delirium [28].

### Assessment of potential predictors

A systematic review of prior studies (eTable 1, supplement 1) and an expert panel of health care professionals (e.g., psychiatrists, geriatricians, geriatric nursing specialists, and pharmacists) were consulted to identify the following predictors for delirium: age, sex, marital status, education, type of admission, smoking, alcohol intake, infection, constipation, sleep, vision, hearing, pain, functional status, cognitive function, drug use, depression, nutritional status, and comorbidities. Among these factors, demographic and general clinical characteristics including age, sex, marital status, education, marital status, smoking, alcohol intake, constipation, sleep, and type of admission, were recorded. Vision or hearing impairment data were extracted from the patient’s medical history and physical diagnosis, as described by the patient, or based on the use of glasses or hearing aids on a regular basis. The patients were termed functionally dependent if their Barthel Index (BI), measuring activities of daily living (ADL), score was less than 100 [29]. The Short Portable Mental Status Questionnaire (SPMSQ) was used to assess cognitive function, and all scores were adjusted by educational level [30]. In addition, we tracked the patient’s medical diagnosis history and information provided by caregivers in terms of cognitive function. To assess nutritional status, the Short Form of Mini-Nutritional Assessment (MNA-SF) was utilized, and malnutrition was defined as a score of 11 [31]. Patients with depression were characterized as having a Geriatric Depression Scale-15 (GDS-15) score greater than 5 [32], and comorbidities were assessed using the Charlson Comorbidity Index (CCI), a score based on 19 chronic diseases [33]. Medication information was obtained from the Hospital Information System of West China Hospital. Polypharmacy was defined as the concurrent use of ≥5 medicines on admission, including both prescription and nonprescription drugs on the pharmacy list [34]. We used the Faces Pain Scale-Revised (FPS-R) to assess pain [35]. Furthermore, adverse events data, such as falls, physical limitations, and length of hospital stay, were collected.

### Data collection process

After obtaining informed consent from the subjects, face-to-face assessments were performed by trained investigators. Research assessors for delirium and predictor assessment were fully trained by consultant psychiatrists and geriatricians. The investigators followed the patients from the time they were admitted (within 24 h) until 14 days later, during their hospitalization. All patients were assessed for clinical delirium by experienced clinical researchers within 24 h of admission and every 48 h for the first 14 days of hospitalization (i.e., the 3rd, 5th, 7th, 9th, 11th, and 13th days) and the day before discharge. In addition, to minimize errors and maximize reliability, well-trained nurses assessed delirium three times daily (8:00 AM~ 18:00 PM, 18:00 PM ~ 2:00 AM the next day, and 2:00 AM~ 8:00 AM) and more frequently if necessary (e.g., after sudden changes in a patient’s behavior, attention, or consciousness). If the patient was diagnosed with delirium or suspected delirium by nurses, the researchers further independently assessed the patient and tracked the patient’s proximate medical records. Within 24 h of admission, the baseline data and all the potential predictors were evaluated by clinical researchers. Health outcome data were collected throughout the hospital stay.

### Statistical analysis

Data were analysed using the SPSS Statistics 21.0 software package (SPSS Inc., Chicago, USA). Numerical data were presented as means and standard deviations (SDs), whereas categorical data were described as proportions. Univariate and multivariate logistic regression analyses were performed to screen for significant risk factors for delirium. We used the logistic regression (Enter) for multivariate analysis, entering all the variables that were considered clinically relevant and that showed a univariate relationship with delirium outcome (*P* < 0.1). Additionally, a multicollinearity diagnostic was performed to assess the validity of the regression model by calculating the values of tolerance (Tol) and the variance inflation factor (VIF). Tol > 0.1 and VIF < 10 indicated that no multicollinearity existed among the dependent variables. The Hosmer-Lemeshow test was performed to assess goodness of fit. Two-sided *P* < 0.05 was considered statistically significant.

## Results

### Characteristic of the study population

**Fig. 1:**
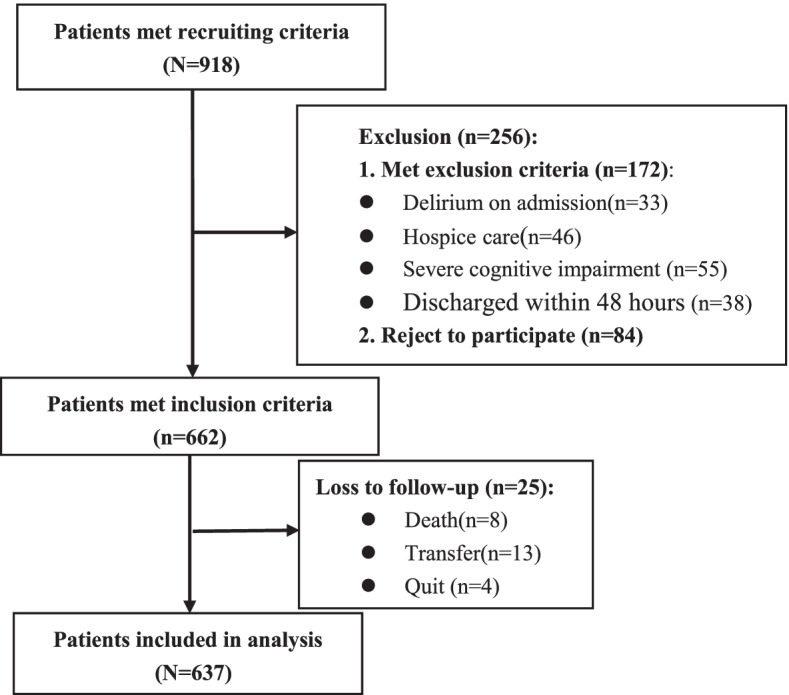
Flow chart of subjects enrolment

Initially, 918 older hospitalized patients in the geriatric department were screened, of whom 281 patients were excluded because of delirium on admission, severe cognitive impairment, end-of-life disease, refusal to participate, discharge within 2 days after admission, or loss to follow-up. The remaining 637 older hospitalized patients satisfied the inclusion criteria (Fig. 1). The ages of the included patients ranged from 80 to 99 years, with a mean age of 85.79 ± 3.69 years, and 464 were males (72.8%). The demographic and baseline characteristics of all the subjects with and without delirium are presented in Table 1.

**Table 1:** Demographic and clinical characteristics of delirium and non-delirium patients

Variables	Total (***N*** = 637)	Delirium (***n*** = 109)	Non-delirium (***n*** = 528)
**Age** (years) **(mean, SD)**	85.79 ± 3.69	86.95 ± 4.21	85.56 ± 3.54
**Sex (n%)**			
Male	464(72.8)	77(70.6)	387(73.3)
Female	173(27.2)	32(29.4)	141(26.7)
**Marital status (n%)**			
Married	512(80.4)	80(73.4)	432(81.8)
Single/divorced /windowed	125(19.6)	29(26.6)	96(18.2)
**Education (n%)**			
Illiteracy/primary school	105(16.5)	26(23.9)	79(15.0)
Middle school	115(18.1)	22(20.2)	93(17.6)
High school/secondary technical	155(24.3)	24(22.0)	131(24.8)
College or above	262(41.1)	37(33.9)	225(42.6)
**Smoking (n%)**			
Yes	390(61.2)	65(59.6)	325(61.6)
No	247(38.8)	44(40.4)	203(38.4)
**Alcohol use (n%)**			
Yes	506(79.4)	80(73.4)	426(80.7)
No	131(20.6)	29(26.6)	102(19.3)
**Number of Medications (mean, SD)**	3.92 ± 2.39	6.25 ± 2.46	3.44 ± 2.07
**CCI (mean, SD)**	1.85 ± 1.80	2.83 ± 2.13	1.64 ± 1.65
**Emergency admission (n%)**			
Yes	81(12.7)	34(31.2)	47(8.9)
No	556(87.3)	75(68.8)	481(91.1)
**Vision (n%)**			
Normal	83(13.1)	17(15.6)	66(12.5)
Impairment	554(86.9)	92(84.4)	462(87.5)
**Hearing (n%)**			
Normal	128(20.1)	20(18.3)	108(20.5)
Impairment	509(79.9)	89(81.7)	420(79.5)
**Sleep (n%)**			
Normal	112(17.6)	26(23.9)	86(13.8)
Impairment	525(82.4)	83(76.1)	442(86.2)
**Pain (n%)**			
Yes	349(54.8)	71(65.1)	278(52.7)
No	288(45.2)	38(34.9)	250(47.3)
**Constipation (n%)**			
Yes	188(29.5)	53(48.6)	135(25.6)
No	449(70.5)	56(51.4)	393(74.4)
**Cognitive function (n%)**			
Normal	404(63.4)	10(9.2)	394(74.6)
Mild cognitive impairment	139(21.8)	38(34.9)	101(19.1)
Moderate cognitive impairment	94(14.8)	61(56.0)	33(6.3)
**ADL (n%)**			
Normal	120(18.8)	13(11.9)	107(20.3)
Impairment	517(81.2)	96(88.1)	421(79.7)
**Nutrition (n%)**			
Normal	390(61.2)	43(39.4)	347(65.7)
Malnutrition	247(38.8)	66(60.6)	181(34.3)
**Depression (n%)**			
Yes	108(17.0)	60(55.0)	48(9.1)
No	529(83.0)	49(45.0)	480(90.9)
**Infection (n%)**			
Yes	145(22.8)	62(56.9)	83(15.7)
No	492(77.2)	47(43.1)	445(84.3)

*Abbreviations*: *CCI* Charlson Comorbidity Index, *ADL* Activity of Daily Life

Data presented as mean ± SD or n (%)

### Incidence and subtypes of delirium

**Fig. 2:**
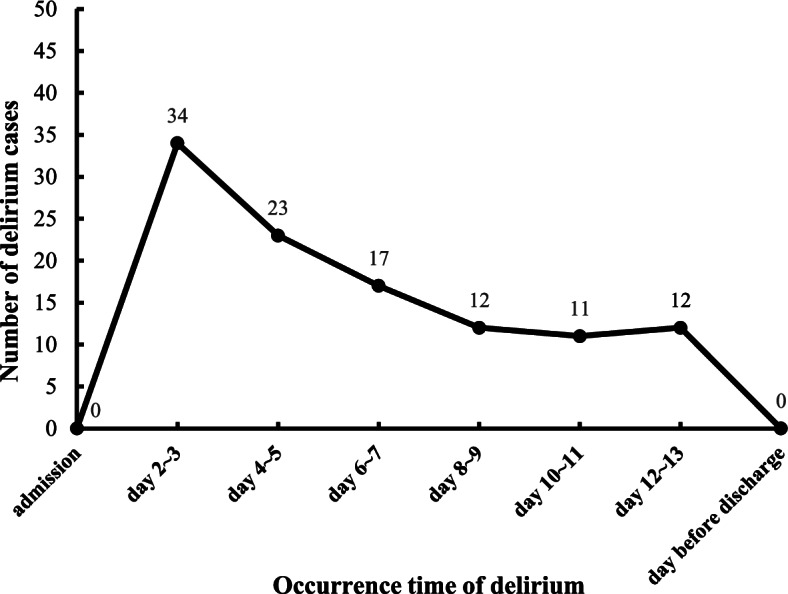
Occurrence time distribution of delirium (*n* = 109)

Of the 637 recruited individuals, a total of 109 patients developed delirium according to the 3D-CAM assessment, with an incidence of 17.1%. After admission, 34 cases occurred on day 2 or day 3 of hospitalization, accounting for 31.2%, and 23 cases occurred on day 4 or day 5 (21.1%). The number of delirium cases on the 6th to 7th, 8th to 9th, 10th to 11th, 12th to 13th days of hospitalization were 17 (15.6%), 12 (11.0%), 11 (10.1%), and 12 (11.0%), respectively (Fig. 2). Among the patients with delirium, 48 (44.1%) had hypoactive delirium, 33.9% (37/109) had hyperactive delirium, and 22.0% (24/109) had mixed delirium.

### Potential risk factors for delirium according to the univariate logistic regression analysis

The univariate logistic regression analysis showed that age, marital status, education, number of medications ≥5, emergency admission, pain, constipation, cognitive function impairment, ADL impairment, malnutrition, depression, infection and CCI score were significantly different between the incident delirium and no delirium groups (*P* < 0.05), as shown in Table 2. The prevalence rates of these risk factors were shown in eTable 2 in supplement 2.

**Table 2:** Univariate logistic regression analyses of potential risk factors for delirium

Variables	Unadjusted OR	95% CI	***P-value***
**Age (90 ~ 99 y)**	2.41	1.47–3.95	< 0.001**
**Sex (Female)**	1.14	0.72–1.80	0.571
**Single/divorced /windowed**	1.63	1.01–2.63	0.045*
**High school or above**	0.61	0.40–0.93	0.023*
**Smoking**	1.08	0.71–1.65	0.708
**Alcohol use**	1.51	0.94–2.44	0.088
**Number of medications ≥ 5**	7.99	5.09–12.54	< 0.001**
**Emergency admission**	4.64	2.81–7.68	< 0.001**
**Vision impairment**	1.29	0.72–2.31	0.383
**Hearing impairment**	1.14	0.67–1.94	0.618
**Sleep deprivation**	1.61	0.98–2.64	0.061
**Pain**	1.68	1.09–2.58	0.018*
**Constipation**	2.76	1.80–4.21	< 0.001**
**Cognitive impairment**	29.11	14.76–57.41	< 0.001**
**ADL impairment**	1.88	1.01–3.48	0.045*
**Malnutrition**	2.94	1.93–4.50	< 0.001**
**Depression**	12.25	7.58–19.79	< 0.001**
**Infection**	7.07	4.53–11.04	< 0.001**
**CCI**			
Mild(≤2)	Reference		
Moderate(3–4)	2.30	1.42–3.74	0.001*
Severe(≥5)	5.29	2.66–10.54	< 0.001**

*Abbreviations*: *ADL* Activity of Daily Life, *CCI* Charlson Comorbidity Index, *OR* odds ratio, *95% CI* 95% confidence interval

* *P* < 0.05; ** *P* < 0.001

### Independent predictors for delirium according to the multivariable logistic regression analysis

Multivariable binary logistic regression was performed, entering all variables with *P* < 0.1 in the univariate analysis and variables that were considered clinically relevant. In addition, the multicollinearity diagnosis showed that the VIF was < 2 and the Tol was > 0.7, indicating that there was no obvious multicollinearity among the included factors. The binary variables were assigned (0,1), and the dummy variables were set for the multiclassification variables. Then, they were all entered into the regression model. Finally, the multivariable binary logistic regression analysis showed that factors that remained independently associated with delirium were cognitive function impairment [OR 17.42, 95% CI: (7.47–40.64)], depression [OR 9.30, 95% CI: (4.59–18.84)], CCI ≥5 [OR 4.21, 95% CI: (1.48–12.01)], sleep deprivation [OR 3.89, 95% CI: (1.71–8.82)], infection [OR 3.33, 95% CI: (1.70–6.54)], polypharmacy (number of medications ≥5) [OR 2.85, 95% CI: (1.51–5.39)], constipation [OR 2.58, 95% CI: (1.33–5.02)], and emergency admission [OR 2.13, 95% CI: (1.02–4.45)], as shown in Table 3. The predictive model was fitted by the Hosmer-Lemeshow goodness-of-fit test, with an *x*^2^ value 6.686 and a *P* value of 0.571, showing that the model fit the data well.

**Table 3:** Multivariable binary logistic regression of risk factors for delirium

Variables	B	SE	Adjusted OR (95% CI)	***P-*** value
Age (90–99 y)	0.767	0.408	2.15 (0.968–4.793)	0.060
Single/divorced /windowed	0.167	0.391	1.18 (0.549–2.543)	0.670
High school or above	−0.505	0.328	0.60 (0.317–1.147)	0.123
Alcohol use	0.017	0.375	1.01 (0.488–2.119)	0.964
Polypharmacy (number of medications ≥5)	1.048	0.325	2.85 (1.509–5.389)	0.001
Emergency admission	0.758	0.375	2.13 (1.023–4.452)	0.043
Sleep deprivation	1.357	0.418	3.89 (1.712–8.818)	0.001
Vision impairment	0.589	0.508	1.80 (0.665–4.882)	0.247
Hearing impairment	0.289	0.458	1.34 (0.544–3.276)	0.528
Pain	0.129	0.340	1.14 (0.584–2.214)	0.705
Constipation	0.948	0.339	2.58 (1.327–5.015)	0.005
Cognitive function impairment	2.858	0.432	17.42 (7.469–40.640)	< 0.001
Malnutrition	0.146	0.340	1.16 (0.594–2.255)	0.667
Depression	2.230	0.360	9.30 (4.589–18.844)	< 0.001
ADL impairment	0.745	0.575	2.11 (0.682–6.505)	0.195
CCI: Mild (≤2)			Reference	0.019
CCI: Moderate (3–4)	0.494	0.386	1.64 (0.770–3.489)	0.200
CCI: Severe (≥5)	1.437	0.535	4.21 (1.476–12.006)	0.007
Infection	1.204	0.344	3.33 (1.697–6.542)	< 0.001
Constant	−6.195	0.664		< 0.001

*Abbreviations*: *ADL* Activity of Daily Life, *CCI* Charlson Comorbidity Index, *SE* Standard Error, *OR* odds ratio, *95% CI* 95% confidence interval

### Clinical outcomes

Patients with delirium had significantly longer hospital lengths of stay than those without delirium (mean ± SD, 24.76 ± 11.31 vs. 17.71 ± 8.15, *P* < 0.001). Higher rates of adverse events, such as physical restraint use (6.4% vs. 0%, *P* < 0.001) and falls (3.7% vs. 0%, *P* = 0.001), were observed among the patients who developed delirium.

## Discussion

The principal finding of this prospective study was that 17.1% of hospitalized patients aged 80 years and over had delirium. The independent predictors of delirium in these individuals were cognitive function impairment, depression, comorbidities, sleep deprivation, infection, polypharmacy, constipation, and emergency admission. Additionally, patient with delirium were more likely to have longer hospital stays and higher rates of physical restraint use and falls than those without delirium. To the best of our knowledge, this is the first study directed at very old hospitalized patients, and it represents by far the largest delirium-related study in this population to date.

Previous research reported delirium prevalence in 29% ~ 64% of older medical patients [36], which was higher than that in our sample. It is probable that variances in study populations, as well as diverse delirium diagnostic procedures used by researchers, contributed to the disparity in the delirium occurrence results. In this study, more patients were diagnosed with the hypoactive subtype than the other two subtypes. Similarly, Morandi et al. [37] reported that hypoactive delirium occurred most frequently in older patients.

The strongest predictors of delirium in our study were cognitive impairment (OR 17.42) and depression (OR 9.30). Preexisting cognitive dysfunction has been shown to be not only the most important independent risk factor for delirium but also one of the predictors of persistent delirium [14, 16, 18, 38]. Moreover, delirium can cause long-term cognitive decline and increase the risk of new dementia [39]. We assumed that cholinergic deficiency and activation of the immune system might be the main mechanisms by which dementia patients develop delirium. As cognitive impairment is an important risk factor, it is significantly better to evaluate this condition using a validated and reliable tool rather than only a medical history. Accordingly, in our study, cognitive impairment was assessed by the patient’s medical history, information provided by caregivers, and the validated assessment tool. Approximately 36.6% of our cohort had mild or moderate cognitive impairment. This result is consistent with that of a study that reported the occurrence of cognitive impairment among the senior population was 3 to 42% [40]. The high prevalence of cognitive impairment among the senior population and the fact that it could lead to delirium highlights the need to delay cognitive decline and prevent delirium whenever possible.

Depression was another strong independent predictor. We discovered that depression was present in 17.0% of our cohort and that older individuals with depression were 9.3 times more likely to develop delirium than those without depression. This finding is consistent with earlier research. A systematic review found that older hospitalized patients with depression had a 1.3- to 9.0-fold higher incidence of delirium than nondepressed patients [41]. Furthermore, some research suggests that delirium and depression may have similar pathophysiological mechanisms, and approximately 5% of older hospitalized patients fulfil the diagnostic criteria for both [42]. However, a large-scale epidemiologic study documenting the co-occurrence of both conditions is lacking. Given the risk factors for delirium, older people with major depressive disorder can be treated with pharmacologic or nonpharmacological therapy in conjunction with cognitive therapies [43]. To prevent delirium, we recommend that older inpatients remain intellectually, physically, and socially engaged in life.

Additionally, comorbidities and polypharmacy were also independent risk factors for delirium (comorbidities: OR 4.21; polypharmacy: OR 2.85), consistent with previous research [11, 13, 15, 18]. This is because a very old person with multimorbidity may be unable to resist repeated exposures to multiple stressors, such as hospitalization or surgery, increasing the likelihood of developing delirium. Furthermore, older individuals with multiple chronic conditions commonly receive polypharmacy. In our study cohort, approximately 28.7% of older patients had multimorbidity, and 38.8% of individuals received polypharmacy. Therefore, the drug list for an elderly patient, especially with multiple chronic diseases, should be carefully evaluated, and medications that have been demonstrated to cause delirium should be replaced.

In addition to the demographic and clinical risk factors, we found that environmental factors, such as emergency admission, increased the risk of incident delirium [OR 2.13, 95% CI: (1.02–4.45)]. A total of 12.7% of patients in the current study were admitted to the emergency department (ED) before being transferred to the geriatric department, with an average duration of 16 h. Similarly, Bo et al. discovered an association between the length of ED stay and the development of delirium [44]. This finding may be attributable to the characteristics of ED, such as overcrowding, excess noise, delayed transfer to the ward of admitted individuals, or medication administration [45, 46]. These stressors could induce acute stress responses, which is a widely held hypothesis for the pathophysiology of delirium. Therefore, the incidence of delirium may reflect exposure to more serious environmental factors and greater vulnerability of old adults. The hospital environment may be a target for delirium prevention measures along with delirium intervention programs [47].

In contrast with prior studies that demonstrated an increased risk of delirium associated with older age [13, 16, 48], our study found no age difference in delirium incidence (OR 2.15, *P* = 0.060). However, even though the multivariable logistic regression analysis showed no difference between the age groups (80 ~ 89 years; 90 ~ 99 years), the univariate logistic regression analysis showed a significant difference (*P* < 0.001). Possible reasons for these discrepant may be the age cut-off (≥ 80 years) in our sample, the narrow age range of those included, or the limited number of included patients. Our findings indicated that advanced age was a potential risk factor for incident delirium in very old adults rather than an independent predictor. An interaction of age with other precipitating factors is needed for the development of delirium. Furthermore, we did not find that alcohol consumption or smoking were independent predictors of delirium [49]. We suspect that this is because alcohol withdrawal has characteristics associated with delirium, whereas occasional alcohol usage may have minimal influence on delirium occurrence [50]. Although the patients in our study had a history of smoking or alcohol consumption, the majority of them had been smoke-free or alcohol-free for several years. Hence, the dose–response effect of alcohol or cigarette consumption on the occurrence of delirium requires further investigation.

We also analysed the clinical outcomes of delirium in very old hospitalized individuals. Patients with delirium experienced worse outcomes than patients without delirium, such as longer lengths of stay and higher rates of falls and physical restrictions use. These findings were consistent with prior research findings that linked delirium to an increased risk of adverse outcomes, and another study reported that delirium was the primary risk factor for geriatric falls [19, 36, 51].

The strength of the present study was that it was the first to identify the incidence of delirium in older Chinese patients admitted to medical wards. Previous studies targeted only a single disease or included only those admitted to a single specialized unit. Although Yam et al. reported the prevalence of delirium among older Chinese medical inpatients, incident cases of delirium were not included [24]. Furthermore, we concentrated on very old individuals (aged ≥80 years) who had multiple chronic diseases, polypharmacy, and diminished physiological reserves, making them more vulnerable and at higher risk of developing delirium than those aged less than 80 years [52]. There were some limitations to this study that should be mentioned. The main limitation is that the study covered only medical patients over the age of 80 years, making it difficult to generalize the results to younger or surgical patients. Furthermore, because it was conducted in a single hospital, the results may not be applicable to other clinical settings or communities. Moreover, we tracked the clinical outcomes of patients with and without delirium during their hospitalization, and additional follow-up after discharge is required.

## Conclusion

In conclusion, delirium was common in very elderly medical inpatients and was associated with adverse health outcomes of longer hospital stays and higher rates of physical restraint use and falls. The strongest independent predictors for delirium were cognitive impairment and depression. Other predictors, such as a CCI score ≥ 5, sleep deprivation, infection, polypharmacy, constipation, and emergency admission, all contributed to incident delirium among these individuals. Given these predictors, which enable the early identification of high-risk patients by healthcare providers, targeted preventative actions could be implemented in a timely manner.

## Supplementary Information

**Additional file 1:** **eTable 1.** Literature characteristics on delirium risk prediction models in older medical patients.

**Additional file 2:** **eTable 2.** The prevalence of delirium risk factors as determined by univariate analysis.

## Data Availability

There are no linked research data sets for this paper. Data will be made available on reasonable request to the corresponding author.

## Abbreviations

ICU: Intensive care unit
MMSE: Mini-Mental State Examination
3D-CAM: 3-min Diagnose Interview for CAM-defined Delirium
BI: Barthel Index
ADL: Activity of Daily Life
SPMSQ: Short Portable Mental Status Questionnaire
MNA-SF: Short Form of Mini-Nutritional Assessment
GDS-15: Geriatric Depression Scale-15
CCI: Charlson Comorbidity Index
FPS-R: Faces Pain Scale-Revised
SD: Standard deviation
VIF: Variance Inflation Factor
Tol: Tolerance value
CI: Confidence interval
OR: Odds ratio
ED: Emergency Department

## References

[1] American Psychiatric Association (2013). Diagnostic and statistical manual of mental disorders (DSM-5).

[2] Inouye SK, Westendorp RG, Saczynski JS (2014). Delirium in elderly people. Lancet..

[3] Van den Boogaard M, Schoonhoven L, van der Hoeven JG, van Achterberg T, Pickkers P (2012). Incidence and short-term consequences of delirium in critically ill patients: a prospective observational cohort study. Int J Nurs Stud.

[4] Veiga D, Luis C, Parente D, Fernandes V, Botelho M, Santos P, Abelha F (2012). Postoperative delirium in intensive care patients: risk factors and outcome. Rev Bras Anestesiol.

[5] Hosie A, Davidson PM, Agar M, Sanderson CR, Phillips J (2013). Delirium prevalence, incidence, and implications for screening in specialist palliative care inpatient settings: a systematic review. Palliat Med.

[6] Witlox J, Eurelings LS, de Jonghe JF, Kalisvaart KJ, Eikelenboom P, van Gool WA (2010). Delirium in elderly patients and the risk of post-discharge mortality, institutionalization, and dementia: a meta-analysis. JAMA..

[7] Gleason LJ, Schmitt EM, Kosar CM, Tabloski P, Saczynski JS, Robinson T, Cooper Z, Rogers SO, Jones RN, Marcantonio ER, Inouye SK (2015). Effect of delirium and other major complications on outcomes after elective surgery in older adults. JAMA Surg.

[8] Leslie DL, Marcantonio ER, Zhang Y, Leo-Summers L, Inouye SK (2008). One-year health care costs associated with delirium in the elderly population. Arch Intern Med.

[9] National Clinical Guideline Centre (UK). Delirium: Diagnosis, Prevention and Management [Internet]. London: Royal College of Physicians (UK); 2010. PMID: 22319805. 22319805

[10] Hshieh TT, Yue J, Oh E (2015). Effectiveness of multicomponent nonpharmacological delirium interventions: a meta-analysis. JAMA Intern Med.

[11] Inouye SK, Charpentier PA (1996). Precipitating factors for delirium in hospitalized elderly persons. Predictive model and interrelationship with baseline vulnerability. JAMA..

[12] Inouye SK (2006). Delirium in older persons. N Engl J Med.

[13] Caeiro L, Ferro JM, Albuquerque R, Figueira ML (2004). Delirium in the first days of acute stroke. J Neurol.

[14] Inouye SK, Viscoli CM, Horwitz RI, Hurst LD, Tinetti ME (1993). A predictive model for delirium in hospitalized elderly medical patients based on admission characteristics. Ann Intern Med.

[15] Inouye SK, Zhang Y, Jones RN, Kiely DK, Yang F, Marcantonio ER (2007). Risk factors for delirium at discharge: development and validation of a predictive model. Arch Intern Med.

[16] Korevaar JC, van Munster BC, de Rooij SE (2005). Risk factors for delirium in acutely admitted elderly patients: a prospective cohort study. BMC Geriatr.

[17] Pompei P, Foreman M, Cassel CK, Alessi C, Cox D (1995). Detecting delirium among hospitalized older patients. Arch Intern Med.

[18] Pompei P, Foreman M, Rudberg MA, Inouye SK, Braund V, Cassel CK (1994). Delirium in hospitalized older persons: outcomes and predictors. J Am Geriatr Soc.

[19] Chaiwat O, Chanidnuan M, Pancharoen W, Vijitmala K, Danpornprasert P, Toadithep P, Thanakiattiwibun C (2019). Postoperative delirium in critically ill surgical patients: incidence, risk factors, and predictive scores. BMC Anesthesiol.

[20] Dai YT, Lou MF, Yip PK, Huang GS (2000). Risk factors and incidence of postoperative delirium in elderly Chinese patients. Gerontology..

[21] Miu DK, Yeung JC (2013). Incidence of post-stroke delirium and 1-year outcome. Geriatr Gerontol Int.

[22] Miu DK, Chan CW, Kok C (2016). Delirium among elderly patients admitted to a post-acute care facility and 3-months outcome. Geriatr Gerontol Int.

[23] Qu J, Chen Y, Luo G, Zhong H, Xiao W, Yin H (2018). Delirium in the acute phase of ischemic stroke: incidence, risk factors, and effects on functional outcome. J Stroke Cerebrovasc Dis.

[24] Yam KK, Shea YF, Chan TC, Chiu KC, Luk JK, Chu LW, Chan FH (2018). Prevalence and risk factors of delirium and subsyndromal delirium in Chinese older adults. Geriatr Gerontol Int.

[25] Martinez JA, Belastegui A, Basabe I, Goicoechea X, Aguirre C, Lizeaga N, Urreta I, Emparanza JI (2012). Derivation and validation of a clinical prediction rule for delirium in patients admitted to a medical ward: an observational study. BMJ Open.

[26] Marcantonio ER, Ngo LH, O'Connor M, Jones RN, Crane PK, Metzger ED, Inouye SK (2014). 3D-CAM: derivation and validation of a 3-minute diagnostic interview for CAM-defined delirium: a cross-sectional diagnostic test study. Ann Intern Med.

[27] Gao LL, Xie DM, Dong BR, Yue JR (2018). The validity and reliability of the Chinese version of 3D-CAM for the detection of delirium in the elderly. Chin J Geriatr.

[28] Huang M-C, Lee C-H, Lai Y-C, Kao Y-F, Lin H-Y, Chen C-H (2009). Chinese version of the delirium rating scale-revised-98: reliability and validity. Compr Psychiatry.

[29] Mahoney FJ, Barthel DW (1965). Functional evaluation: the Barthel index. Maryland Medical Journal.

[30] Pfeiffer E (1975). A short portable mental status questionnaire for the assessment of organic brain deficit in elderly patients. J Am Geriatr Soc.

[31] Kaiser MJ, Bauer JM, Ramsch C, Uter W, Guigoz Y, Cederholm T, Thomas DR, Anthony P, Charlton KE, Maggio M, Tsai AC, Grathwohl D, Vellas B, Sieber CC (2009). MNA-international group. validation of the mini nutritional assessment short-form (MNA-SF): a practical tool for identification of nutritional status. J Nutr Health Aging.

[32] Nyunt MS, Fones C, Niti M, Ng TP (2009). Criterion-based validity and reliability of the geriatric depression screening scale (GDS-15) in a large validation sample of community-living Asian older adults. Aging Ment Health.

[33] Charlson ME, Pompei P, Ales KL, MacKenzie CR (1987). A new method of classifying prognostic comorbidity in longitudinal studies: development and validation. J Chronic Dis.

[34] Gnjidic D, Hilmer SN, Blyth FM, Naganathan V, Waite L, Seibel MJ, McLachlan AJ, Cumming RG, Handelsman DJ, Le Couteur DG (2012). Polypharmacy cutoff and outcomes: five or more medicines were used to identify community-dwelling older men at risk of different adverse outcomes. J Clin Epidemiol.

[35] Hicks CL, von Baeyer CL, Spafford PA, van Korlaar I, Goodenough B (2001). The faces pain scale-revised: toward a common metric in pediatric pain measurement. Pain..

[36] Hshieh TT, Inouye SK, Oh ES (2020). Delirium in the elderly. Clin Geriatr Med.

[37] Morandi A, Di Santo SG, Cherubini A, Mossello E, Meagher D, Mazzone A, Bianchetti A, Ferrara N, Ferrari A, Musicco M, Trabucchi M, Bellelli G, ISGoD group (2017). Clinical features associated with delirium motor subtypes in older inpatients: results of a multicenter study. Am J Geriatr Psychiatry.

[38] Ahmed S, Leurent B, Sampson EL (2014). Risk factors for incident delirium among older people in acute hospital medical units: a systematic review and meta-analysis. Age Ageing.

[39] Fong TG, Inouye SK, Jones RN (2017). Delirium, Dementia, and Decline. JAMA Psychiatry.

[40] Tricco AC, Soobiah C, Lillie E, Perrier L, Chen MH, Hemmelgarn B, Majumdar SR, Straus SE (2012). Use of cognitive enhancers for mild cognitive impairment: protocol for a systematic review and network meta-analysis. Syst Rev.

[41] O'Sullivan R, Inouye SK, Meagher D (2014). Delirium and depression: inter-relationship and clinical overlap in elderly people. Lancet Psychiatry.

[42] Givens JL, Jones RN, Inouye SK (2009). The overlap syndrome of depression and delirium in older hospitalized patients. J Am Geriatr Soc.

[43] Siu AL, Bibbins-Domingo K, Grossman DC, Baumann LC, Davidson KW, Ebell M, García FA, Gillman M, Herzstein J, Kemper AR, Krist AH, Kurth AE, Owens DK, Phillips WR, Phipps MG, Pignone MP, US Preventive Services Task Force (USPSTF) (2016). Screening for depression in adults: US preventive services task force recommendation statement. JAMA..

[44] Bo M, Bonetto M, Bottignole G, Porrino P, Coppo E, Tibaldi M, Ceci G, Raspo S, Cappa G, Bellelli G (2016). Length of stay in the emergency department and occurrence of delirium in older medical patients. J Am Geriatr Soc.

[45] Hastings SN, Schmader KE, Sloane RJ, Weinberger M, Goldberg KC, Oddone EZ (2007). Adverse health outcomes after discharge from the emergency department--incidence and risk factors in a veteran population. J Gen Intern Med.

[46] Carpenter CR, Platts-Mills TF (2013). Evolving prehospital, emergency department, and “inpatient” management models for geriatric emergencies. Clin Geriatr Med.

[47] Evensen S, Saltvedt I, Lydersen S, Wyller TB, Taraldsen K, Sletvold O (2018). Environmental factors and risk of delirium in geriatric patients: an observational study. BMC Geriatr.

[48] Sharma A, Malhotra S, Grover S, Jindal SK (2012). Incidence, prevalence, risk factor and outcome of delirium in intensive care unit: a study from India. Gen Hosp Psychiatry.

[49] Ouimet S, Kavanagh BP, Gottfried SB, Skrobik Y (2007). Incidence, risk factors and consequences of ICU delirium. Intensive Care Med.

[50] Awissi DK, Lebrun G, Coursin DB, Riker RR, Skrobik Y (2013). Alcohol withdrawal and delirium tremens in the critically ill: a systematic review and commentary. Intensive Care Med.

[51] Mazur K, Wilczyński K, Szewieczek J (2016). Geriatric falls in the context of a hospital fall prevention program: delirium, low body mass index, and other risk factors. Clin Interv Aging.

[52] Douglas VC, Hessler CS, Dhaliwal G, Betjemann JP, Fukuda KA, Alameddine LR, Lucatorto R, Johnston SC, Josephson SA (2013). The AWOL tool: derivation and validation of a delirium prediction rule. J Hosp Med.

