# Supplementary material for: Incidence, predictors and health outcomes of delirium in very old hospitalized patients: a prospective cohort study
Source: BMC Geriatr. 2022 Mar 29;22:262. doi: 10.1186/s12877-022-02932-9 (PMC8966247; doi:10.1186/s12877-022-02932-9)
Supplement: Supplementary file 1 — Additional file 1: eTable 1. Literature characteristics on delirium risk prediction models in older medical patients. [file 12877_2022_2932_MOESM1_ESM.pdf]

e Table 1 Literature characteristics on delirium risk prediction models in older medical patients

Author	Subjects	No. of Sample	Delirium incidence	Study Design	Model Statistics	Predictors
Sharon K. et al., 1993[1]	Medical patients, age>70	281	25%	Prospective cohort study	proportional hazards model	Visual impairment, serious illness, cognitive impairment, blood urea, nitrogen/creatinine>18
Pompei et al., 1994[2]	Medical hospitalized elderly patients, age≥65	432	15%	Prospective cohort study	Logistic regression	Cognitive impairment, disease burden, depression, alcoholism
O'keefe ST et al., 1996[3]	Acute Care Unit Patients	225	28%	Prospective cohort study	Logistic regression	Dementia, serious illness, elevated blood urea levels
Inouye SK. et al.,1996[4]	hospitalized elderly patients, age>70	408	18%	Prospective cohort study	proportional hazards model	Use of physical restraint, malnutrition, more than 3 drugs, placement of urinary catheter, hospital-related adverse events
Inouye SK. et al., 2007[5]	Medical patients, age≥70	491	11.8%	Prospective cohort study	Logistic regression	Dementia, visual impairment, functional decline, Charlson score, use constraints
Jouko VL. et al., 2008[6]	Frail hospitalized elderly patient with delirium, age≥70	87	None	Cross-sectional study	Logistic regression	Infections, metabolic abnormalities, drug side effects, cardiovascular and cerebrovascular accidents
Rudolph JL et al., 2011[7]	Age>65	110	23%	Prospective cohort study	proportional hazards model	Cognitive impairment, sensory disturbances, severe illness, blood urea nitrogen/creatinine>18
Martinez JA et al., 2012[8]	Medical ward patients, age>18	699	13%	Prospective cohort study	Logistic regression	Age>85, ADL deficiency, antipsychotics
Kobayashi D et al., 2013[9]	Medical ward patients	3570	3.8%	Retrospective cohort study	Logistic regression, CHAID decision tree	Logistic regression: delirium history, ADL deficiency, malignancy, alcoholism, dementia;<br>CHAID model: delirium history, age, malignancy, ADL deficiency
Douglas VC et al., 2013[10]	emergency medical patients, age≥50	374	12% (8.5%)	Prospective cohort study	Logistic regression	Age>80, Inability to spell WORLD backwards (language impairment), disorientation, serious illness
Carraso MP	Medical ward	478	6.7%	Prospective	Logistic	Self-care deficits,

Author	Subjects	No. of Sample	Delirium incidence	Study Design	Model Statistics	Predictors
et al., 2014[11]	patients, age≥65		（11.5%）	cohort study	regression	blood urea nitrogen/creatinine>18
Otremba I et al., 2016[12]	Geriatric medical ward patient, age 60~100	675	7.99%	Cross-sectional survey	Logistic regression	Transfer, dementia, history of delirium, history of falls, use of proton pump inhibitors
Foroughan M et al., 2016[13]	Internal medicine hospitalized elderly patients, age>60	200	22%	Cross-sectional survey	Logistic regression	hemoglobin≤12, blood urea nitrogen/creatinine≥1/20, C-reactive protein positive, depression, cognitive decline
de Wit HA . et al., 2016[14]	Delirium patient, age≥60	646	17.4%	Prospective cohort study	Logistic regression	Age, polypharmacy, hypoglycemic side effects, cholinergic insufficiency, dopamine overdose, antipsychotics, pain relievers, analgesics, sleeping pills
Kim H et al., 2016 [15]	Medical and post-operative hospitalized patients; age 68.9±13.0; 54.3±16.8	337	None	Retrospective cohort study	Logistic regression	Delirium group: lower plasma albumin, hypertension, mechanical ventilation, antipsychotic drug use. Postoperative delirium: Stroke history, hypertension, ICU care, medication

References:

1) Inouye SK, Viscoli CM, Horwitz RI, Hurst LD, Tinetti ME. A predictive model for delirium in hospitalized elderly medical patients based on admission characteristics. Ann Intern Med. 1993;15;119(6):474-81.
2) Pompei P, Foreman M, Rudberg MA, Inouye SK, Braund V, Cassel CK. Delirium in hospitalized older persons: outcomes and predictors. J Am Geriatr Soc. 1994;42(8):809-15.
3) O'Keeffe ST, Lavan JN. Predicting delirium in elderly patients: development and validation of a risk-stratification model. Age Ageing. 1996;25(4):317-21.
4) Inouye SK, Charpentier PA. Precipitating factors for delirium in hospitalized elderly persons. Predictive model and interrelationship with baseline vulnerability. JAMA. 1996 20;275(11):852-7
5) Inouye SK, Zhang Y, Jones RN, Kiely DK, Yang F, Marcantonio ER. Risk factors for delirium at discharge: development and validation of a predictive model. Arch Intern Med. 2007;9;167(13):1406-13.
6) Laurila JV, Laakkonen ML, Tilvis RS, Pitkala KH. Predisposing and precipitating factors for delirium in a frail geriatric population. J Psychosom Res. 2008;65(3):249-54.
7) Rudolph JL, Harrington MB, Lucatorto MA, Chester JG, Francis J, Shay KJ; Veterans Affairs and Delirium Working Group. Validation of a medical record-based delirium risk assessment. J Am Geriatr Soc. 2011;59 Suppl 2(Suppl 2):S289-94.

8) Martinez JA, Belastegui A, Basabe I, Goicoechea X, Aguirre C, Lizeaga N, Urreta I, Emparanza JI. Derivation and validation of a clinical prediction rule for delirium in patients admitted to a medical ward: an observational study. BMJ Open. 2012 ;14;2(5):e001599.
9) Kobayashi D, Takahashi O, Arioka H, Koga S, Fukui T. A prediction rule for the development of delirium among patients in medical wards: Chi-Square Automatic Interaction Detector (CHAID) decision tree analysis model. Am J Geriatr Psychiatry. 2013;21(10):957-62.
10) Douglas VC, Hessler CS, Dhaliwal G, Betjemann JP, Fukuda KA, Alameddine LR, Lucatorto R, Johnston SC, Josephson SA. The AWOL tool: derivation and validation of a delirium prediction rule. J Hosp Med. 2013;8(9):493-9.
11) Carrasco MP, Villarroel L, Andrade M, Calderón J, González M. Development and validation of a delirium predictive score in older people. Age Ageing. 2014;43(3):346-51.
12) Otremba I, Wilczyński K, Szewieczek J. Delirium in the geriatric unit: proton-pump inhibitors and other risk factors. Clin Interv Aging. 2016 ; 4;11:397-405.
13) Foroughan M, Delbari A, Said SE, AkbariKamrani AA, Rashedi V, Zandi T. Risk factors and clinical aspects of delirium in elderly hospitalized patients in Iran. Aging Clin Exp Res. 2016;28(2):313-9.
14) de Wit HA, Winkens B, Mestres Gonzalvo C, Hurkens KP, Mulder WJ, Janknegt R, Verhey FR, van der Kuy PH, Schols JM. The development of an automated ward independent delirium risk prediction model. Int J Clin Pharm. 2016;38(4):915-23. 15.
15) Kim H, Chung S, Joo YH, Lee JS. The major risk factors for delirium in a clinical setting. Neuropsychiatr Dis Treat. 2016;21;12:1787-93.
