# Supplementary material for: Incidence, predictors and health outcomes of delirium in very old hospitalized patients: a prospective cohort study
Source: BMC Geriatr. 2022 Mar 29;22:262. doi: 10.1186/s12877-022-02932-9 (PMC8966247; doi:10.1186/s12877-022-02932-9)
Supplement: Supplementary file 2 — Additional file 2: eTable 2. The prevalence of delirium risk factors as determined by univariate analysis. [file 12877_2022_2932_MOESM2_ESM.pdf]

eTable 2 The prevalence of delirium risk factors as determined by univariate analysis

Variables	Prevalence rate (%)
Age (90~99 years)	15.4
Single/divorced /windowed	19.6
High school/secondary technical college or above	65.4
Number of medications ≥5	38.8
Emergency admission	12.7
Pain	54.8
Sleep deprivation	82.4
Constipation	29.5
Cognitive impairment	36.6
ADL Impairment	81.2
Malnutrition	38.8
Depression	17.0
Infection	22.8
Comorbidities	28.7
